# Randomised trial of cord clamping and initial stabilisation at very preterm birth

**DOI:** 10.1136/archdischild-2016-312567

**Published:** 2017-09-18

**Authors:** Lelia Duley, Jon Dorling, Angela Pushpa-Rajah, Sam J Oddie, Charles William Yoxall, Bernard Schoonakker, Lucy Bradshaw, Eleanor J Mitchell, Joe Anthony Fawke

**Affiliations:** 1 Nottingham Clinical Trials Unit, Queen’s Medical Centre, University of Nottingham, Nottingham, UK; 2 Early Life Research Group, Queen’s Medical Centre, University of Nottingham, Nottingham, UK; 3 Department of Dermatology, Guy’s Hospital, London, UK; 4 Centre for Reviews and Dissemination, Hull York Medical School, University of York, Heslington, York, UK; 5 Neonatal Unit, Liverpool Women’s Hospital, Liverpool, UK; 6 Neonatal Intensive Care Unit, City Hospital, Nottingham, UK; 7 Neonatal Unit, Leicester Royal Infirmary, Leicester, UK

**Keywords:** preterm birth, randomised trial, cord clamping, neonatal care with umbilical cord intact, intraventricular haemorrhage

## Abstract

**Objectives:**

For very preterm births, to compare alternative policies for umbilical cord clamping and immediate neonatal care.

**Design:**

Parallel group randomised (1:1) trial, using sealed opaque numbered envelopes.

**Setting:**

Eight UK tertiary maternity units.

**Participants:**

261 women expected to have a live birth before 32 weeks, and their 276 babies.

**Interventions:**

Cord clamping after at least 2 min and immediate neonatal care with cord intact, or clamping within 20 s and immediate neonatal care after clamping.

**Main outcome measures:**

Intraventricular haemorrhage (IVH), death before discharge.

**Results:**

132 women (137 babies) were allocated clamping ≥2 min and neonatal care cord intact, and 129 (139) clamping ≤20 s and neonatal care after clamping; six mother-infant dyads were excluded (2, 4) as birth was after 35^+6^ weeks, one withdrew (death data only available) (0, 1). Median gestation was 28.9 weeks for those allocated clamping ≥2 min, and 29.2 for those allocated clamping ≤20 s. Median time to clamping was 120 and 11 s, respectively. 7 of 135 infants (5.2%) allocated clamping ≥2 min died and 15 of 135 (11.1%) allocated clamping ≤20 s; risk difference (RD) −5.9% (95% CI −12.4% to 0.6%). Of live births, 43 of 134 (32%) had IVH vs 47 of 132 (36%), respectively; RD −3.5% (−14.9% to 7.8%). There were no clear differences in other outcomes for infants or mothers.

**Conclusions:**

This is promising evidence that clamping after at least 2 min and immediate neonatal care with cord intact at very preterm birth may improve outcome; a large trial is urgently needed.

**Trial registration:**

ISRCTN 21456601.

**What is already known on this topic?**

- If the umbilical cord is not clamped flow continues for longer than previously thought, and cord pulsation does not correlate with umbilical flow.
- A short delay in cord clamping may reduce the risk of intraventricular haemorrhage and improve outcome in preterm babies.
- Previous small trials excluded babies requiring resuscitation at birth.

**What this study adds?**

- Neonatal stabilisation and resuscitation can be provided with the cord intact.
- Giving neonatal care with the cord intact may improve outcome for the infants born very preterm.
- The effect of resuscitating preterm babies with an intact cord needs to be addressed in a large multicentre trial.

## Introduction

At birth, if the umbilical cord is not clamped blood flow between baby and placenta may continue for several minutes.^1–3^ This umbilical flow is part of the physiological transition from the fetal to the neonatal circulation; clamping the cord too soon may restrict the infant’s ability to cope with this transition.^2 4–8^ A short delay in cord clamping may increase neonatal blood volume, a longer delay of several minutes may have more substantive advantages, such as smoother cardiorespiratory transition and more stable blood pressure, which for very preterm infants (<32 weeks gestation) may reduce the risk of intraventricular haemorrhage.^9 10^ Concerns about deferring (delaying) cord clamping include exacerbating jaundice, polycythaemia, delayed respiratory support and hypothermia.

The relevant Cochrane review suggests that for infants born preterm deferring cord clamping may improve outcomes at hospital discharge.^11^ However, the 15 trials included were small and have a high risk of bias. None were prospectively registered, and they reported different outcomes, so the possibility of selective outcome reporting is high. Also, data on long-term safety are sparse. Importantly, these studies excluded infants requiring resuscitation at birth, and for very preterm births most trials deferred clamping for <60 s. Guidelines for very preterm birth make various recommendations about when to clamp the cord,^12–14^ and advise that clamping should not be delayed if neonatal resuscitation is required.^14 15^ Evaluating timing of cord clamping at very preterm birth is a research priority, particularly for infants requiring resuscitation at birth.^15–17^

Our hypothesis is that for very preterm births a policy of cord clamping after at least 2 min and providing immediate neonatal care with cord intact, rather than usual care of clamping within 20 s and neonatal care after clamping, improves outcome for the infants without adverse effects for the women. Having developed strategies for providing stabilisation and resuscitation with the cord intact,^18 19^ we conducted a randomised trial.

## Methods

### Trial design and changes since trial registration

This pragmatic randomised parallel group trial was conducted at eight UK tertiary maternity units with a neonatal intensive care unit. The protocol is published^20^ along with an update,^21^ and these are summarised here. Initially, the study aimed to assess the feasibility of a large multicentre trial and planned to recruit for 1 year. Feasibility was demonstrated and, on the advice of the independent Trial Steering Committee, recruitment continued while funding was sought for the main trial. Recruitment closed in February 2015 when the funding application was unsuccessful. The sponsor is Nottingham University Hospitals NHS Trust. Trial coordination was at the Nottingham Clinical Trials Unit (NCTU).

As the original protocol was for a pilot trial, outcomes were measures of feasibility and analysis by allocated group was not planned. The statistical analysis plan (SAP) for the extended study was agreed before data were unblinded. For the planned main trial, main outcomes were death before discharge and intraventricular haemorrhage (all grades), hence these were the main outcomes in this SAP. Data presented here are for outcomes at discharge. Follow-up for women at 1 year, and for children at age 2 years (corrected for gestation) will be reported separately.

### Parent and parent representative involvement

Three parent representatives were coinvestigators, involved in identifying the research question, securing funding and designing and conducting the study. They advised on plans for recruitment; contributed to developing the oral assent pathway; advised on study implementation and conduct; participated in investigator meetings, including training in explaining the study to women and offering consent and contributed to interpretation and writing up of results. Two parents were independent members of the Trial Steering Committee. Participants in the trial have been thanked for participation, asked for their views of participation and updated on progress. When results are published they will be summarised in lay language.

### Participants

Women were eligible if they were expected to have a live birth before 32 weeks gestation, regardless of mode of birth or fetal presentation. Exclusion criteria were monochorionic twins; triplets or higher-order multiple pregnancy and known major congenital malformation.

### Interventions

Umbilical cord clamping after at least 2 min and, if needed, immediate neonatal stabilisation and resuscitation with cord intact was compared with clamping within 20 s and, if needed, immediate neonatal stabilisation and resuscitation after clamping. Waiting at least 2 min before clamping was based on a balance between waiting until umbilical flow ceased (which may take 3–5 min, or longer),^2 3 8 22^ and what was acceptable to clinicians. For the intervention group, with cord intact babies were placed onto a firm surface with easy access to resuscitation equipment; either the usual equipment^19^ or a smaller trolley designed for this purpose^23^ moved alongside the mother’s bed. For caesarean births, the neonatal resuscitation equipment was covered with sterile drapes, and the neonatologist scrubbed and gowned. For both groups, after cord clamping neonatal care was either beside the mother or at the usual location (side of the room or separate room), at the discretion of the local clinicians. Six sites used their usual resuscitation equipment (153 women recruited) and two the trolley (108). Until cord clamping, the baby was kept at the level of placenta (introitus or mothers’ abdomen, or if a caesarean birth the anterior thigh). Clamping within 20 s with stabilisation and resuscitation after cord clamping was based on the current UK practice,^24^ and previous trials.^11^

For both groups, all other aspects of care including intubation and respiratory support were at the discretion of the attending clinicians. Neonatal care was based on local unit policy and consistent with Resuscitation Council (UK) newborn life support guidelines.^14 25^ Standard equipment was used according to local practice, including plastic sheets or bags, towels and hats, warming mattresses or overhead heaters and saturation monitors.

### Outcome measures

Main outcomes were death before hospital discharge and intraventricular haemorrhage (all grades using the Papile classification^26^). A single assessor reviewed the cranial ultrasound scan reports for intraventricular haemorrhage, blind to the allocated group. Then eight trained clinicians (neonatologists or radiologists) independently adjudicated each scan, blind to allocation.^27^ If the adjudication disagreed with the scan report review, a second independent adjudicator assessed the scan images. Remaining discrepancies were resolved by discussion.

Other outcomes for the baby were: severe intraventricular haemorrhage,^26^ periventricular leukomalacia, blood transfusion, hypothermia (<36°C, <35°C), chronic lung disease (supplemental oxygen or ventilator support at 36 weeks postmenstrual age), ventilation, necrotising enterocolitis (grade 2 or higher), clinical sepsis, treatment for jaundice, treatment for patent ductus arteriosus, treatment for retinopathy of prematurity and duration of hospital stay.

Outcomes for women were: postpartum haemorrhage (≥500 or ≥1000 mL), postpartum infection, breast feeding and for vaginal births manual removal of placenta and third stage of labour longer than 30 min. Data were collected up to discharge by research staff at site.

### Recruitment and randomisation

Study information was available in antenatal clinics and wards. Women at risk of very preterm birth were offered participation, and if they accepted gave written consent. Eligibility and consent were checked before randomisation, which was during labour or before caesarean section. If birth was imminent and the attending clinician considered it appropriate, women were offered a brief explanation of the study and offered participation (oral assent). Those who gave oral assent were then randomised. After the birth, these women had an opportunity to discuss the study and were invited to give written consent for participation in follow-up.

Randomisation was by attending clinicians, who took the next sealed consecutively numbered opaque envelope from a ringbinder folder. Sequence generation (1:1) was by computer, stratified by centre with balanced blocks of randomly varying size, created by NCTU. On the envelope was a label to record the date, time, woman’s initials, her date of birth and gestation. Once this label was completed she was randomised, even if the envelope was not opened. Inside the envelope was a yellow card instructing when to clamp the cord, and a ‘Birth Record’ (plus a second for twins) to record information about the third stage of labour and neonatal care at birth. This was completed by clinical staff, and filed in the baby’s medical notes. Used envelopes and yellow cards were placed in a locked mailbox, which was emptied regularly. Details from each envelope were entered into the online randomisation log maintained by NCTU. If an envelope was taken but not used, it was returned unopened to NCTU.

During the last few months of recruitment, a secure web-based randomisation system, using the same randomisation sequence, was introduced in three sites to assess its feasibility.

### Sample size

Based on a total of 43 600 live births per year at the eight maternity units, we expected 610 (1.4%)^28^ live births to be before 32 weeks; as target accrual was 16%–18% of eligible births we anticipated 100–110 women randomised per year. This was planned as a pilot trial so there was no formal power calculation.

### Statistical analysis

All analyses are based on the groups as randomly allocated (intention to treat). For twin pregnancies, outcome is reported for both babies. Where appropriate, results are presented as relative risk (RR) or risk difference (RD) with 95% CIs. Women who were randomised but gave birth after 35^+6^ weeks are excluded, as outcomes for these babies are different from those born very preterm. For secondary outcomes, missing data for individual items are only reported if they exceeded 1% of total data available. No formal interim analysis was planned. Data were monitored in confidence by an independent Data Monitoring Committee, which met four times.

## Results

**Figure 1:**
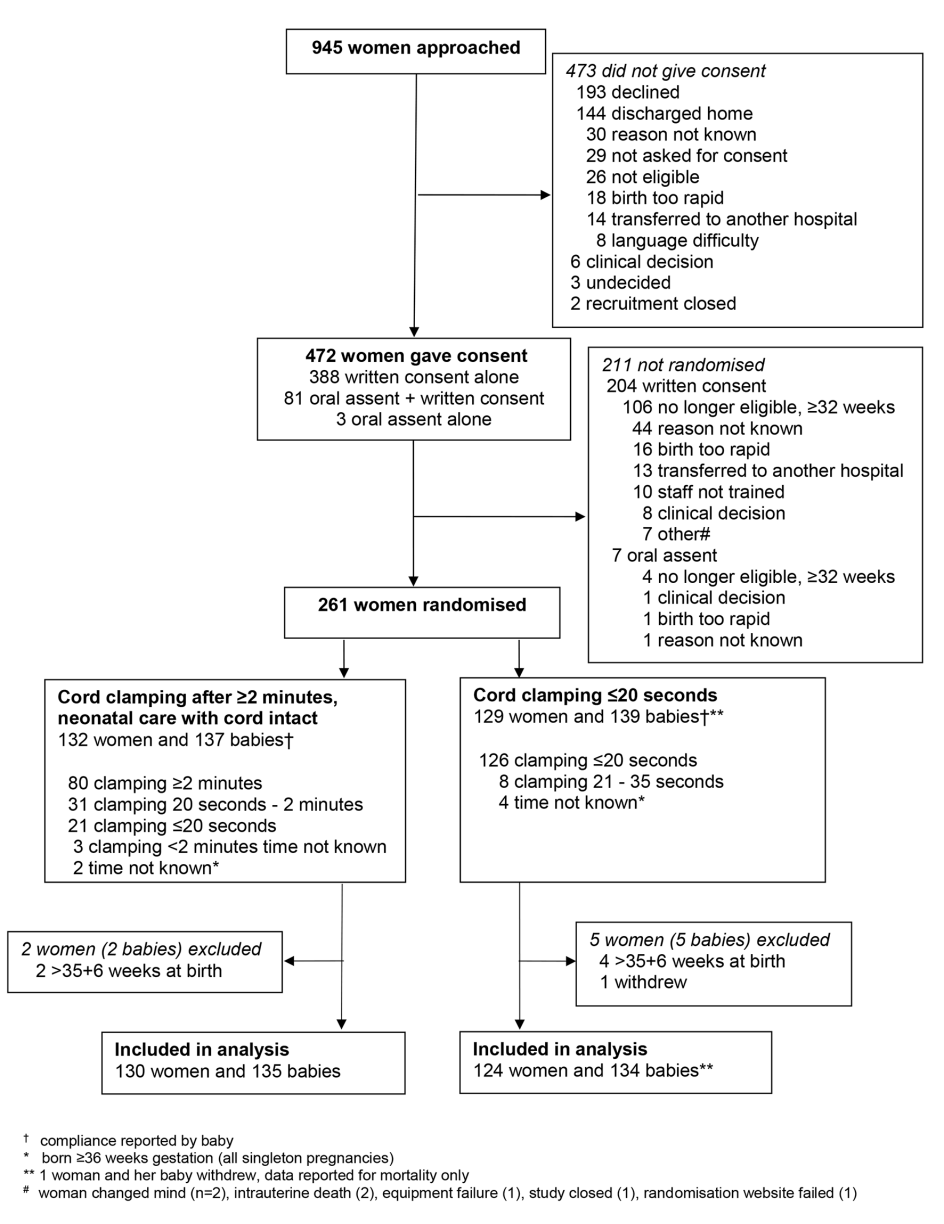
Participant flow.

Of 945 women approached, 472 (50%) gave consent (figure 1). Oral assent was offered to 93 women, of whom 84 (90%) assented and of these 77 were randomised. For eight of the 77 women randomised following oral assent birth was not imminent as anticipated, and written consent was given before birth, hence 69 were randomised with oral assent only (33 cord clamping ≥2 min, 36 clamping ≤20 s). For 66 of these women written consent was given after the birth; written consent was not obtained for three, for two their baby died and they did not return for their counselling appointment at which written consent would have been sought and one was transferred to another unit.

Recruitment was from March 2013 to February 2015, and 261 women were randomised (132 cord clamping after at least 2 min, 129 within 20 s). Six women were excluded as they gave birth after 35^+6^ weeks and one withdrew (outcome data reported only for death before discharge), leaving 254 women for analysis (figure 1). In 2 of the 17 twin pregnancies, one fetus died in utero before randomisation, so 269 babies are included in analysis. Time between randomisation and birth was within 30 min for 81 women (32%), within 1 hour for 128 (50%) and within 2 hours for 178 (70%); 20 (8%) gave birth 24 hours or more after randomisation.

### Baseline characteristics

The groups were balanced at trial entry (table 1). A third of women were randomised before 28 weeks, and two-thirds before 30 weeks. One hundred forty-four (57%) were in their first pregnancy and just over half had a caesarean birth. Of the babies, 143 (53%) were males (71/135 clamping after ≥2 min vs 72/134 clamping ≤20 s).

**Table 1:** Baseline characteristics for the women

	Clamp ≥2 min+neonatal care with cord intact	Clamp ≤20 s+neonatal care after clamping
	(n=130)	(n=124)
Gestation at randomisation (weeks) ≥32	1 (1%)	1 (1%)
30 to 31^+6^	44 (34%)	46 (37%)
28 to 29^+6^	38 (29%)	42 (34%)
26 to 27^+6^	25 (19%)	21 (17%)
<26	22 (17%)	14 (11%)
Gestation at birth (weeks) ≥32	2 (2%)	2 (2%)
30 to 31^+6^	47 (36%)	44 (35%)
28 to 29^+6^	35 (27%)	43 (35%)
26 to 27^+6^	25 (19%)	21 (17%)
<26	21 (16%)	14 (11%)
Age (years), mean (SD)	30.3 (6.1)	29.2 (6.6)
Primiparous	69 (53%)	75 (60%)
Twin pregnancy	*7 (5%)	10 (8%)
Pregnancy complications		
Prelabour rupture of membranes	43 (33%)	40 (32%)
Spontaneous onset of labour	31 (24%)	33 (27%)
CTG abnormalities/fetal distress	21 (16%)	23 (19%)
Pre-eclampsia/PIH	33 (25%)	24 (19%)
Antepartum haemorrhage/placenta previa	14 (11%)	18 (15%)
Chorioamnionitis	15 (12%)	14 (11%)
Fetal growth restriction/small for gestational age	13 (10%)	13 (10%)
Other†	5 (4%)	6 (5%)
In last week received		
Magnesium sulfate	67 (52%)	49 (40%)
Not known	4 (3%)	–
Corticosteroids	117 (90%)	111 (90%)
Caesarean section	82 (63%)	67 (54%)
Before labour	69	57
During labour	13	10
Vaginal birth	48 (37%)	57 (46%)
Breech presentation	9	8

*For two, one twin known intrauterine death before randomisation.

†Clamp ≤20 s: abdominal pain (n=2), severe asthma (1), pyelonephritis (1), antiphosphate lipid syndrome (1) and not known (1). Clamp ≥2 min: renal failure and diabetes (1), previous cervical surgery (1), coagulopathy (1), Crohn’s disease (1) and hypotension (1).

CTG, cardiotocograph; PIH, pregnancy-induced hypertension.

### Compliance with the allocated intervention

**Figure 2:**
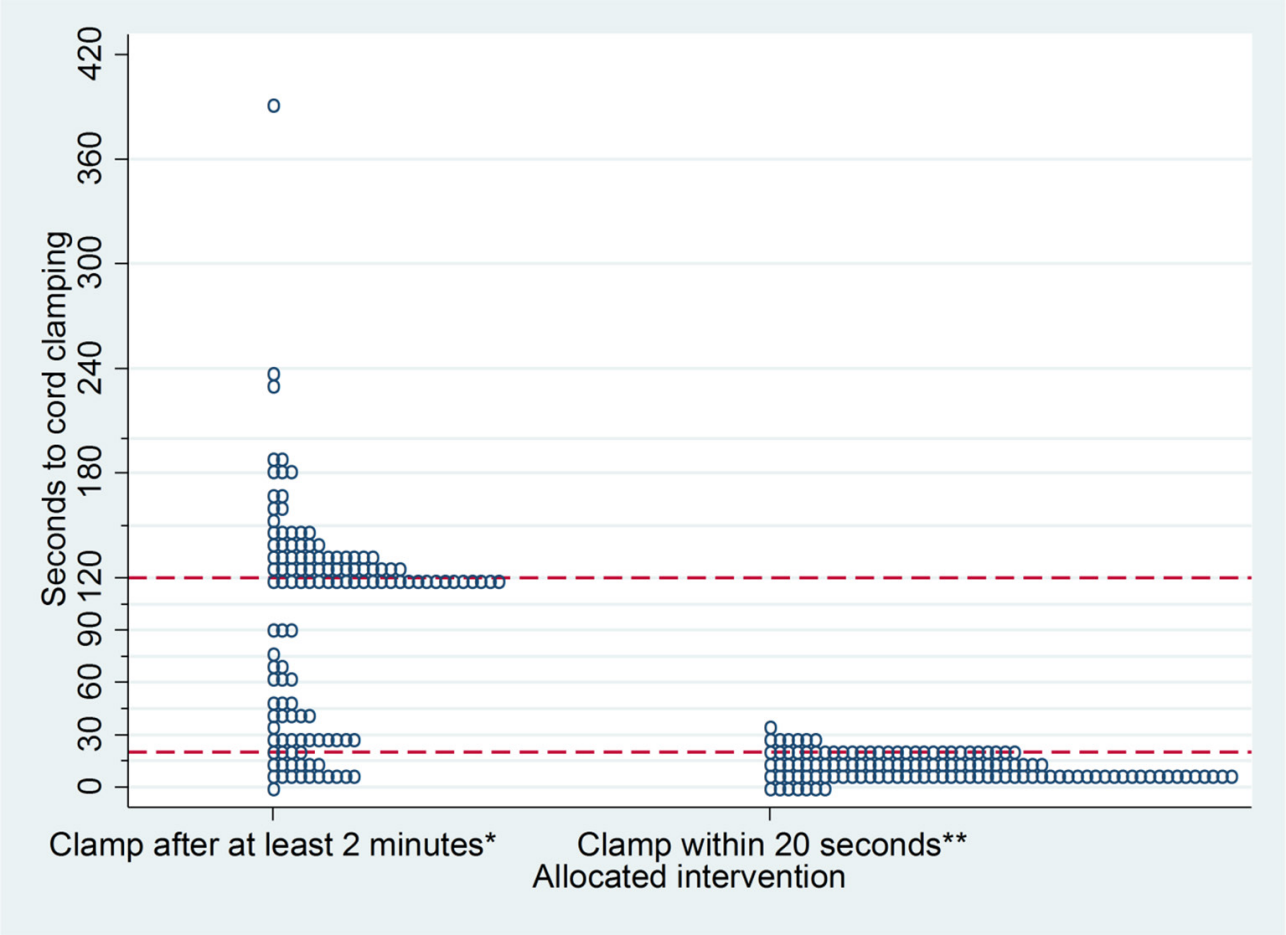
Actual timing of cord clamping for each baby in the two allocated groups. *n=135 babies, seconds to cord clamping not known for 9. **n=134 babies, seconds to cord clamping not known for 9.

Median time to cord clamping was 120 s (IQR 36–134), for those allocated clamping after at least 2 min and neonatal care with cord intact, and 11 s for those allocated clamping within 20 s and neonatal care after clamping (IQR 10–20). In the intervention group, cord clamping was after at least 2 min for 80 (59%) babies and after 20 s for 111 (82%) (figure 2). Baseline characteristics were similar for babies with cord clamping after at least 2 min and those clamped before 2 min; for example, gestation at birth 28^+0^ to 31^+6^ weeks 64% vs 63%, respectively; antenatal corticosteroids 87% vs 94% and caesarean birth 61% vs 67%. For the 55 babies for whom clamping was before 2 min, reasons were: cord too short (21, 38%), an issue that improved with experience; clinical decision (12, 22%); baby born either membranes intact or with the placenta (8, 15%); placental abruption (5, 9%); neonatal team not there in time (4, 7%); staff error (4, 7%) and cord snapped (1, 2%). In the control group, cord clamping was within 20 s for 126 (94%). There were no clear differences in compliance based on whether the site used usual resuscitation equipment or the trolley (data not shown). Neonatal care was comparable between the two allocated groups (table 2). For liveborn babies, median birth weight was 1108 g (IQR 880–1360) in the intervention group and 1180 g (IQR 900–1418 g) in the control group (figure 2).

**Table 2:** Neonatal care and newborn life support at birth

	Clamp ≥2 min+neonatal care with cord intact (n=135)			Clamp ≤20 s+neonatal care after clamping (n=134)		
	Beside mother	Away from mother	Total	Beside mother	Away from mother	Total
Baby in plastic bag/sheet	100*	15	115 (85%)	41*	67	108 (81%)
Mask ventilation	68	32	100 (74%)	14	89	105† (78%)
Supplemental oxygen	46	38	84 (62%)	9	79	89‡ (66%)
Airway suction	39	36	75 (56%)	10	76	87‡ (65%)
Successful intubation	38	40	78 (58%)	10	77	87 (65%)
Surfactant	28	39	67 (50%)	9	66	75 (56%)
Attempted, unsuccessful intubation	20	15	35 (26%)	5	33	38 (28%)
Continuous positive airway pressure	27	17	44 (33%)	–	34	34 (25%)
Cardiac massage	3	3	6 (4%)	3	7	10 (7%)
Umbilical venous catheterisation	1	2	3 (2%)	–	6	6 (4%)
Other	–	1§	1 (1%)	–	–	–

*Placed in plastic bag beside mother, received all other care at roomside; n=14 clamp ≥2 min, n=26 clamp ≤20 s.

†Location not known for two.

‡Location not known for one.

§Packed cell transfusion.

A prophylactic uterotonic drug was given to 127 women (98%) allocated clamping after at least 2 min, and 120 (97%) allocated clamping within 20 s. Administration was before cord clamping for 40 (31%) and 15 (12%), respectively; however, timing was unknown for 47 (37%) and 34 (28%).

### Outcome for babies

Overall, 7/135 (5.2%) babies allocated clamping after at least 2 min and neonatal care with cord intact died compared with 15/135 (11.1%) allocated clamping within 20 s and neonatal care after clamping (table 3), RR 0.47 (95% CI 0.20 to 1.11); RD −5.9% (−12.4% to 0.6%). The only death of an infant born after 30 weeks gestation was due to congenital anomaly that was not known before randomisation (if it had been the woman would not have been eligible). Excluding this death gives RR 0.50 (0.21 to 1.20), and RD −5.2% (-11.5% to 1.2%). The three stillbirths were born before 28 weeks, and were resuscitated at birth; cause of death was intrapartum asphyxia for two, and antepartum infection for one.

**Table 3:** Mortality for the baby before discharge from hospital

	Clamp ≥2 min+neonatal care with cord intact	Clamp ≤20 s+neonatal care after clamping
	(n=135)	(n=135)*
Death	7 (5%)	15 (11%)
Stillbirth	1	2
Early neonatal death	3	7
Late neonatal death	2	5
Postneonatal death	1	1
Gestation at birth (weeks)		
30–31^+6^	–	1
28–29^+6^	1	3
26–27^+6^	–	4
<26	6	7
Cause of death:		
Congenital anomaly†	–	1
Severe pulmonary immaturity	3	3
Intrapartum asphyxia	1	1
IVH	1	2
Infection		
Antepartum	–	1
Early onset	1	1
Late onset	–	2
Necrotising enterocolitis	–	2
Other‡	1	2

*Includes the baby of one woman who withdrew.

Clamp ≥2 min: prolonged oligohydramnios (n=1); clamp ≤20 s: myocardial ischaemia (1), prolonged oligohydramnios (1).

†Not known before randomisation.

Of live births allocated to the intervention, 43/134 (32%) had an IVH versus 47/132 (36%) allocated to the control group (RR 0.90 95% CI 0.64 to 1.26; RD −3.5% (95% CI −14.9% to 7.8%)) (table 4). Overall, 251 babies (94%) had a cranial ultrasound scan (127 vs 124, respectively), and both adjudicated scan and review of the original clinical scan report were available for 224/266 (84%). Diagnosis of IVH was based on adjudication for 81 infants, scan report only for 8 and site data only for 1. There were no clear differences between the allocated groups for any other outcome (table 4).

**Table 4:** Neonatal morbidity for liveborn babies

	Clamp ≥2 min+neonatal care with cord intact	Clamp ≤20 s+neonatal care after clamping
	(n=134)	(n=132)
Any IVH (grade1–4)	43 (32%)	47 (36%)
Alive at discharge from hospital	38	42
Severe IVH (grade 3 or 4)	6 (4%)	7 (5%)
Periventricular leukomalacia	7 (5%)	8 (6%)
Other brain injury	5 (4%)	10 (8%)
Porencephalic cysts	–	1
Ventriculomegaly	–	3
Other*	5	7
Heart rate <100 at 1 min	46† (34%)	49 (37%)
Temperature, admission to NICU (°C) mean (SD)	36.7 (0.6)‡	36.9 (0.8)
≤36°C	17 (13%)	14 (11%)
≤35°C	2 (1%)	3 (2%)
Blood transfusion (any)	63 (47%)	68 (52%)
For anaemia	58	66
For hypotension	–	6
Other indication	4	7
Jaundice requiring treatment	123 (92%)	120 (91%)
Phototherapy	123	120
Exchange transfusion	–	–
Polycythaemia requiring intravenous fluids	–	1 (1%)
Chronic lung disease§	40 (31%)	39 (33%)
Ventilation	100 (75%)	103 (78%)
Duration (days), median (IQR)	3 (1, 9)	2 (1, 9)
Necrotising enterocolitis	8 (6%)	5 (4%)
X-ray with perforation or pneumatosis	7	3
Laparotomy	6	3
Suspected necrotising enterocolitis¶	15 (11%)	13 (10%)
Sepsis	72 (53%)	80 (61%)
Positive culture+antibiotics ≥5 days	30	33
Negative culture+antibiotics ≥5 days	42	47
Treatment for:		
Patent ductus arteriosus	20 (15%)	20 (15%)
Retinopathy of prematurity§	5 (4%)	5 (4%)
Duration of hospital stay** (days) median (IQR)	57 (39, 85)	57 (38, 75)
Mother’s breast milk at discharge**††	71 (55%)	68 (57%)

*Clamp ≥2 min: prominent subarachnoid spaces suggestive of atropy (n=2), periventricular cyst (1), absent cavum septum pellucidum (1), occipital cyst (1). Clamp ≤20 s: prominent subarachnoid spaces suggestive of atropy (n=2), periventricular echodensities (1), increased echogenicity of deep white matter (1), mega cysterna (1), ventriculitis (1) and marked ventricular asymmetry (1).

†Not known n=2.

‡Temperature not recorded for two babies.

§Babies who survived to 36 weeks postmenstrual age: n=129 clamping ≥2 min, n=120 clamping ≤20 s. Data collected at 36 weeks postmenstrual age, or discharge whichever happened first.

¶Defined as bowel rest+antibiotics ≥5 days.

**Admitted to neonatal unit and alive at discharge: n=128 clamping ≥2 min; n=120 clamping ≤20 s.

††Not known n=3 clamping ≥2 min; n=1 clamping ≤20 s.

### Outcome for women

There was no clear difference between the groups in blood loss at birth (≥500 mL 58/130 vs 59/124, RR 0.97, 0.71 to 1.35; ≥1000 mL 11/130 vs 13/124; RR 0.81, 0.38 to 1.73), or in any other outcomes for the women (table 5).

**Table 5:** Outcome for the women

	Clamp ≥2 min+neonatal care with cord intact	Clamp ≤20 s+neonatal care after clamping
	n=130	n=124
Blood loss at birth		
≥500 mL	58 (45%)	59 (48%)
≥1000 mL	11 (8%)	13 (10%)
For vaginal births*		
manual removal of placenta	5 (10%)	6 (11%)
Third stage>30 min	4 (8%)	6 (11%)
Blood transfusion	5 (4%)	3 (2%)
Postpartum infection+parenteral antibiotics	34 (26%)	29 (23%)
Fever >38°C	8 (6%)	5 (4%)
Duration of hospital stay (days) median (IQR)	4 (2, 6)	4 (2, 6)
Expressing/breast feeding at discharge†	121 (96%)	111 (93%)

*n=48 clamping ≥2 min; n=57 clamping ≤20 s.

†For women whose babies were alive at time of their discharge, n=126 clamping ≥2 min; n=120 clamping ≤20 s.

## Discussion

This trial suggests that cord clamping after at least 2 min and providing neonatal care with cord intact may improve outcome at discharge for the infants compared with early clamping and neonatal care after clamping, but the CIs are wide and include harm. We achieved a substantial difference of 109 s between the two groups in median time to clamping, and neonatal care at birth was equivalent in the two groups. For 82% of infants allocated clamping after at least 2 min umbilical flow continued for longer than in the control group, as the cord was clamped after 20 s. Providing neonatal care with cord intact required a multidisciplinary team approach, planning and training. For the neonatal teams, training was consistent with newborn life support recommendations,^14 15^ and included communication with the woman, her partner and other clinical staff. The decision to intubate was based on local hospital policy; the risk of intubation was similar in the two allocated groups, and consistent with the UK practice.^29^ Our study demonstrates that neonatal care with the cord intact is feasible using a range of procedures, and can be done with existing resuscitation equipment^19^ or with a trolley designed for this purpose.^18 23^ This is a more practical and potentially more widely generalisable strategy for supporting continued umbilical flow at birth than in other trials to date.

This study was not powered to detect differences between the allocated interventions. Nevertheless, the most striking result is the difference in death before discharge, this is based on a small number of events however with a wide CI that cannot rule out any difference. Also, there are no clear differences between the groups in IVH or any other serious morbidity that would potentially explain a difference in mortality. Hence, this difference probably reflects the play of chance.

Bringing neonatal care to the mother’s bedside allows her and her partner to share the first moments of their child’s life. Family presence during resuscitation is standard for other areas of healthcare, where it is preferred by families and appears to be beneficial.^30–33^ Our preliminary work suggests neonatal care beside the mother is acceptable to women and their partners,^34^ and to clinicians.^18 35^ Nevertheless, some women reported negative emotions,^34^ and clinicians, particularly those with less experience, were concerned about ‘performing’ in front of the parents.^35^ Few babies in these studies required resuscitation, however, and further evaluation is required.

In our study, median birth weight was lower, rather than higher, for infants allocated deferred clamping; suggesting that net change in neonatal blood volume may not be relevant for very preterm births and supporting our hypothesis that continued umbilical flow has a role in the expanding pulmonary circulation during transition to the neonatal circulation.

### Strengths and limitations of this study

Previous trials have largely excluded infants needing immediate resuscitation at birth.^15^ We developed two strategies that enabled us to recruit these high-risk births. First, providing resuscitation with cord intact as needed allowed infants requiring immediate resuscitation at birth to be included. Second, if birth was imminent leaving insufficient time for the usual consent process, women were offered the opportunity to participate through a two-stage oral assent pathway; a quarter of recruitment used this pathway. The two-stage approach seems acceptable to women and to clinicians.^36^ Other strengths are that the study was multicentre and conducted within existing clinical services, hence widely generalisable to similar settings, and that independent adjudication of cranial ultrasound scans improved reliability in ascertainment of IVH.^37^

Although the largest preterm cord clamping trial published to date, this trial was not powered to demonstrate clinically important differences in outcome between the two policies, therefore the key limitation is sample size.

### Comparison with other studies

One previous trial comparing alternative policies for timing of cord clamping (46 babies) has described providing neonatal care with the cord intact,^38^ but the only outcome reported was neonatal blood volume. Other trials comparing alternative policies for cord clamping do not report providing immediate neonatal care with cord intact. One recent trial (150 babies) has evaluated ventilation with cord intact for infants born before 32 weeks gestation, but in this study cord clamping was at 60 s in both intervention arms.^39^

The Cochrane review includes 15 trials (738 babies) before 37 weeks gestation.^11^ Restricting this to trials largely recruiting before 32 weeks and excluding those evaluating cord milking leaves 12 trials (552 babies) with deferred clamping between 30 and 120 s. In these trials, 8/236 (3.4%) babies allocated deferred clamping died compared with 14/250 (5.6%) allocated immediate clamping, and IVH was 31/195 (16%) vs 50/199 (25%), respectively. These event rates are substantially lower than in our trial, as high-risk infants requiring resuscitation at birth were not recruited. In our study, the overall mortality at discharge from hospital (8.1%) is comparable to that reported for infants of a similar gestation admitted to the 37 UK neonatal units participating in the Vermont Oxford Network (for 1116 inborn infants born between 24 and 31 weeks gestation during 2015, mortality at discharge was 10.8%).^40^ This provides reassurance that we successfully recruited a generalisable group of babies. Diagnosis of any IVH (34%) is higher than reported in the UK for similar babies (27%),^40^ but reassuringly severe IVH is similar (5% and 6%, respectively). Hence, the difference in any IVH diagnosis is likely to be due to variation in the grade 1 and 2 IVH, with another potential factor being differences between hospitals in the proportions of infants born before 32 weeks gestation who have a cranial ultrasound scan. Although some previous studies reported that cranial ultrasound scans were conducted blind to the allocation, none have reported independent adjudication of the scan diagnosis.

Other issues with the trials included in the Cochrane Review are that only two appear to have been registered. This may be relevant since most studies reported mortality, the definition (eg, including or excluding major congenital abnormality and/or stillbirth) varied, and of 11 other outcomes none were reported by more than 6 out of the 15 trials: therefore, selective outcome reporting must be at least a possibility. The apparently high compliance noted in some previous trials seems rather implausible and raises the possibility of unreported postrandomisation exclusions, and hence further potential for bias.

Resuscitation guidelines state ’there is insufficient evidence to recommend an appropriate time for clamping the cord in infants who are severely compromised at birth, and that for infants requiring resuscitation, resuscitative intervention remains the immediate priority’.^15^ Clearly, uncertainty remains about when to clamp the cord for infants requiring resuscitative interventions. For preterm infants who are stable at birth and do not require resuscitation, recommendations are to wait at least 30 s and no longer than 3 min.^15 41^

Cord milking is advocated to increase neonatal blood volume without needing to defer cord clamping.^42^ This technique disrupts umbilical blood flow, however, over-riding autoregulation of blood volume and blood pressure. As a different intervention to cord clamping, with greater potential to do harm, it requires rigorous evaluation.^43–45^

## Conclusions

A large multicentre trial is urgently needed to confirm whether a long delay (2 min or more) in cord clamping combined with stabilisation and/or resuscitation with cord intact, rather than standard care, really does improve outcome for very preterm births. This study demonstrates that such a trial is feasible.
